# A benzene-degrading nitrate-reducing microbial consortium displays aerobic and anaerobic benzene degradation pathways

**DOI:** 10.1038/s41598-018-22617-x

**Published:** 2018-03-14

**Authors:** Siavash Atashgahi, Bastian Hornung, Marcelle J. van der Waals, Ulisses Nunes da Rocha, Floor Hugenholtz, Bart Nijsse, Douwe Molenaar, Rob van Spanning, Alfons J. M. Stams, Jan Gerritse, Hauke Smidt

**Affiliations:** 1 Wageningen University & Research, Laboratory of Microbiology, Stippeneng 4, 6708 WE Wageningen, The Netherlands; 2 Wageningen University & Research, Laboratory of Systems and Synthetic Biology, Stippeneng 4, 6708 WE Wageningen, The Netherlands; 3 Deltares, Subsurface and Groundwater Systems, Daltonlaan 600, 3584 BK Utrecht, The Netherlands; 4 Vrije Universiteit Amsterdam, Department of Molecular Cell Biology, De Boelelaan 1108, 1081 HZ Amsterdam, The Netherlands; 5 Department of Environmental Microbiology, Helmholtz Centre for Environmental Research - UFZ, Leipzig, Germany; 6 Centre of Biological Engineering, University of Minho, Braga, Portugal

## Abstract

In this study, we report transcription of genes involved in aerobic and anaerobic benzene degradation pathways in a benzene-degrading denitrifying continuous culture. Transcripts associated with the family *Peptococcaceae* dominated all samples (21–36% relative abundance) indicating their key role in the community. We found a highly transcribed gene cluster encoding a presumed anaerobic benzene carboxylase (AbcA and AbcD) and a benzoate-coenzyme A ligase (BzlA). Predicted gene products showed >96% amino acid identity and similar gene order to the corresponding benzene degradation gene cluster described previously, providing further evidence for anaerobic benzene activation via carboxylation. For subsequent benzoyl-CoA dearomatization, *bam*-like genes analogous to the ones found in other strict anaerobes were transcribed, whereas gene transcripts involved in downstream benzoyl-CoA degradation were mostly analogous to the ones described in facultative anaerobes. The concurrent transcription of genes encoding enzymes involved in oxygenase-mediated aerobic benzene degradation suggested oxygen presence in the culture, possibly formed via a recently identified nitric oxide dismutase (Nod). Although we were unable to detect transcription of Nod-encoding genes, addition of nitrite and formate to the continuous culture showed indication for oxygen production. Such an oxygen production would enable aerobic microbes to thrive in oxygen-depleted and nitrate-containing subsurface environments contaminated with hydrocarbons.

## Introduction

Benzene is an important component of petroleum. It easily dissolves in water, but is one of the least reactive aromatic hydrocarbons and a potential human carcinogen^1^. Benzene can be readily degraded aerobically, however, anaerobic benzene degradation is challenging^2^. Lacking potentially destabilizing or reactive substituents, the benzene molecule is thermodynamically very stable especially under anoxic conditions^3^. Both aerobic and anaerobic degradation pathways include benzene activation and channeling towards key intermediates (catechol in aerobic and benzoyl-CoA in anaerobic pathways), the upper pathway for dearomatization and ring cleavage and the lower pathway for generation of tricarboxylic acid cycle intermediates (reviewed in^2,4–6^). The genes and enzymes involved in anaerobic benzene activation are not well-studied^7^. Three putative reactions have been proposed for anaerobic benzene activation: hydroxylation to phenol^8–11^, direct carboxylation to benzoate^8,12–15^ and methylation to toluene^16^.

In contrast to many aerobic benzene-degrading pure cultures, only few anaerobic benzene-degrading axenic cultures have been described. The hyperthermophilic archaeon *Ferroglobus placidus* was proposed to employ a putative UbiD-related carboxylase in anaerobic benzene activation^17^, and anaerobic benzene oxidation in *Geobacter metallireducens* was shown to proceed via hydroxylation to phenol^18,19^. In contrast to these strictly anaerobic iron-reducers that employ oxygen-independent activation routes, the chlorate-reducing *Alicycliphilus denitrificans* strain BC^20^ was shown to degrade benzene via an oxygenase-mediated pathway^21^. Such ‘intra-aerobic’ anaerobes apparently derive oxygen species from inorganic oxo-compounds such as nitrate or chlorate for classical aerobic degradation of hydrocarbons^22–25^. The nitrate-reducing facultatively anaerobic *Dechloromonas*^26^ may recruit enzymes of a yet unknown pathway for initial benzene activation^27^. This hypothesis is based on the finding that the genome of *Dechloromonas aromatica* strain RCB lacks the genes involved in anaerobic degradation of monoaromatic compounds whereas it contains genes for their aerobic activation, including several mono- and dioxygenases^28^. Moreover, the oxygen incorporated into benzene to produce phenol by this bacterium does not originate from water^9^ whereas the oxygen source for anaerobic metabolism of benzene to phenol is water^11^. The benzene degradation pathways of the nitrate-reducing *Azoarcus* strains^29^ have not been investigated in details.

Due to the limited availability of anaerobic benzene-degrading isolates, mixed microbial communities were predominantly studied to reveal the physiology and phylogeny of anaerobic benzene degraders and potential anaerobic benzene activation genes and mechanisms^12,15,29–42^. Among different microbial communities involved in anaerobic benzene degradation, members of the strictly anaerobic *Peptococcaceae* (*Clostridiales*) were prevalently found in enrichment cultures with different electron acceptors and proposed as the key players in the initial steps of benzene degradation^12,30–32,36,38,40–42^. Among these studies, two cultures were suggested to activate benzene via carboxylation^41,42^. A proteogenomic analysis using a benzene-degrading iron-reducing enrichment culture identified a putative benzene degradation gene cluster^41^. The products of the putative benzene carboxylase genes (AbcAD) were specifically detected in cultures growing on benzene but not in those growing on phenol or benzoate, suggestive for their role in initial benzene carboxylation^41^. A metatranscriptomic analysis using nitrate-reducing enrichment cultures showed high levels of transcripts of the proposed benzene carboxylation genes (*abcAD*, *bzlA*)^42^. Also in this case, these high levels were seen only in benzene-amended cultures but not in benzoate-fed cultures^42^.

In this study our aim was to elucidate anaerobic benzene degradation using a nitrate-reducing continuous enrichment culture growing for more than 15 years. A former DNA-stable isotope probing (SIP) study with ^13^C-labeled-benzene identified *Peptococcaceae* as the predominant members involved in initial benzene degradation^38^. Efforts to isolate benzene-degrading members of the *Peptococcaceae* have failed, likely because they require syntrophic interactions with partner species. Recent microbial community analysis using Illumina MiSeq next generation technology sequencing (NGS) and quantitative PCR (qPCR) showed high (relative) abundance of the *Peptococcaceae* 16S ribosomal RNA (rRNA) gene and *abcA* gene, further supporting the role of *Peptococcaceae* in benzene degradation via initial carboxylation^40^. Here, we performed a metatranscriptomic study using the same enrichment culture. Our results are in line with the former studies on benzene carboxylation by *Peptococcaceae*^41,42^ corroborating the concept that carboxylation initiates benzene degradation in the absence of oxygen. The observed downstream pathway involved in further breakdown of the benzoate mostly resembled that of facultative anaerobes. Interestingly, transcripts of genes involved in oxygenase-mediated aerobic benzene degradation were also identified.

## Results and Discussion

In the present study, we aimed to elucidate benzene degradation pathways in an anaerobic continuous biofilm culture that was initially inoculated with soil from a benzene-polluted industrial location and enriched for years with benzene as substrate and nitrate as the electron acceptor. The culture was shown to be dominated by Gram-positive *Peptococcaceae*-related microorganisms^38,40^. We here conducted a metatranscriptomic analysis of this microbial consortium to track transcripts involved in anaerobic benzene degradation. We analyzed six samples in our transcriptomic study obtained from two types of biofilm morphologies growing in the reactor: four samples containing white biofilm (samples 1–4) and two samples containing brown biofilm (samples 5–6) (Table S1). After rRNA depletion, cDNA synthesis and sequencing using the Illumina HiSeq platform, a total of 83,662,373 reads was initially obtained with rRNA reads ranging between 0.3–6.9% (Table S2).

### Active community members

**Figure 1:**
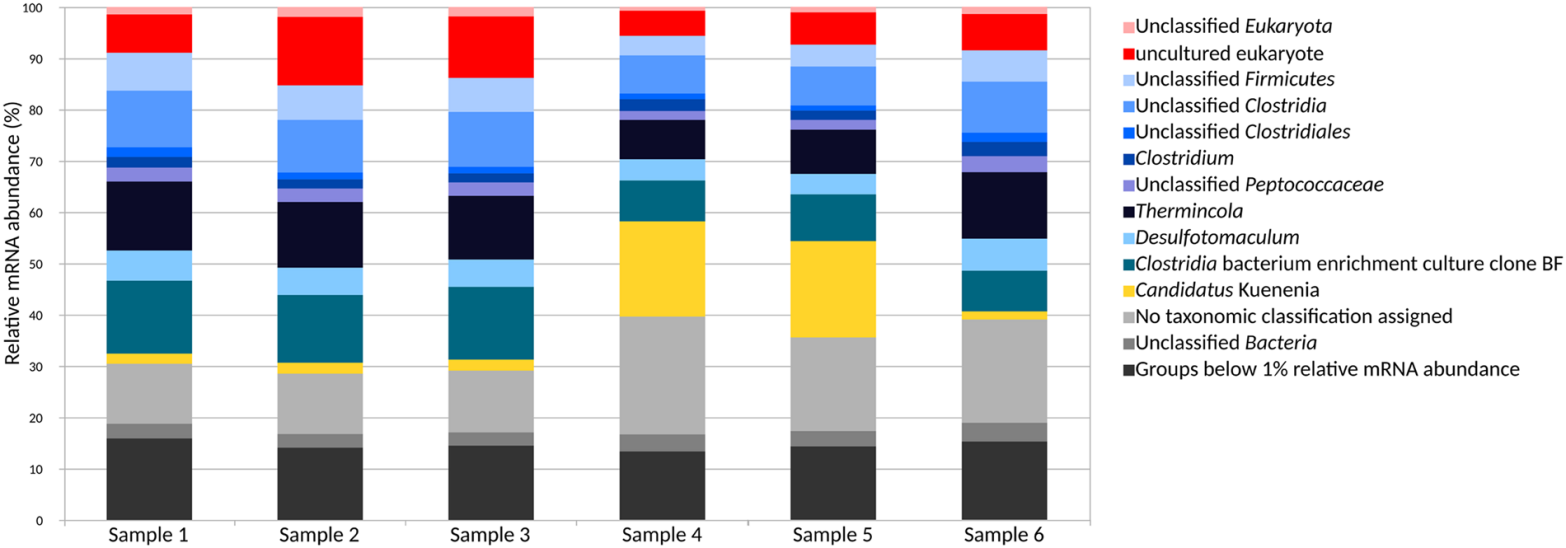
Taxonomic comparison of active microbial communities at mRNA level. Samples 1–4 are from white biofilms and sample 5–6 are from the brown biofilms.

Diverse microbial groups were found in the transcriptome dataset even though the continuous culture was running for more than 15 years (Fig. 1). This could be due to the presence of scavengers growing on dead biomass and cheaters that do not directly contribute to benzene degradation^36^. The transcripts associated with strictly anaerobic *Firmicutes* dominated all samples with 36–59% relative abundance (Fig. 1). Among these were high levels of transcripts assigned to members of the *Peptococcaceae* (21–36% relative abundance). In line with former reports, this suggests a key role of *Peptococcaceae* in anaerobic benzene degradation^12,30–32,36,38,40–42^. The transcripts assigned to Candidatus *Kuenenia* (*Planctomycetes*) were found at a higher relative abundance in samples 4–5 (Table S1). In our previous microbial biofilm community analysis using DNA-SIP with ^13^C-labeled benzene and 16S rRNA gene clone libraries, members of the phyla *Firmicutes* (37% of clones) and *Planctomycetes* (28% of clones) dominated the libraries^38^. In contrast, *Planctomycetes* were not among the most predominant community members in our recent phylogenetic analysis at DNA-level using MiSeq sequencing of PCR-amplified partial 16S rRNA genes^40^. In turn, members of the families *Anaerolineaceae*, *Rhodocyclaceae*, *Comamonadaceae* and SJA-28 were identified as predominant community members^40^, but not in the metatranscriptomic analysis described here. Discrepancy between abundance and activity of microbes has been described previously^43–45^.

### Transcription of genes involved in anaerobic benzene degradation

**Figure 2:**
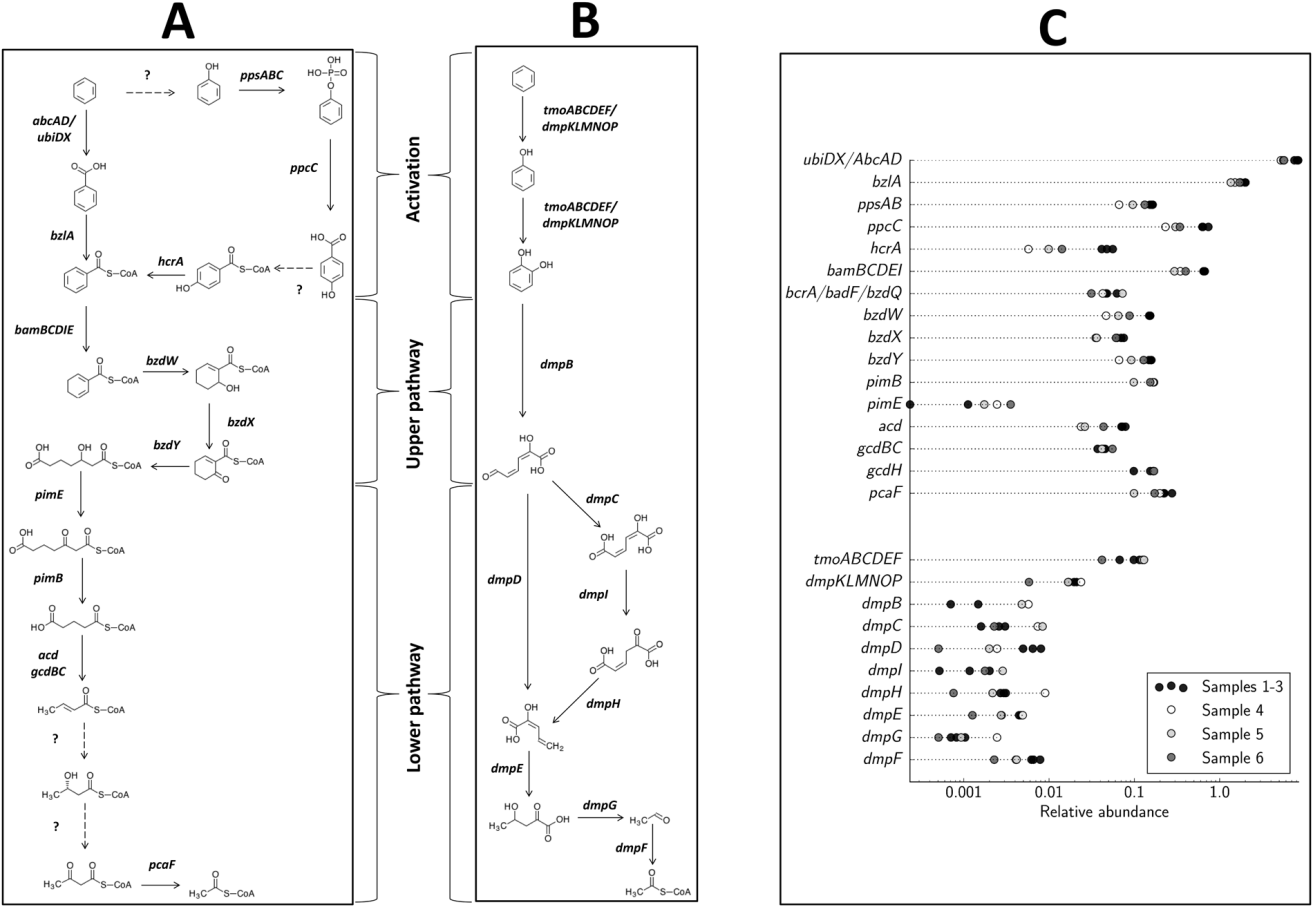
Gene transcripts identified in reactor samples corresponding to known or hypothesized enzymes involved in anaerobic (**A**) and aerobic (**B**) benzene degradation in different microbes and their relative abundances (%) (**C**). Gene transcripts that could not be distinguished due to overlapping assignment with similar genes in the pathway are shown with question marks (full list is given in Table 1). Note that only the substrate and products of each enzymatic reaction are given for clarity. The bar showing the number of relative abundance was log scaled and 0 values were removed.

As described in more detail in the following sections, we found transcription of genes potentially involved in anaerobic benzene activation and subsequent pathways for further degradation of the initially formed benzoyl-CoA (Fig. 2A,C, Table 1).

**Table 1:** Summary of transcribed genes predicted to be involved in anaerobic and aerobic benzene degradation. First column lists the transcribed genes (based on the order of genes in Fig. 2C) followed by the locus tag of each gene. The third column shows the taxonomy of the locus tag, based on megablast/blastn hits of the whole contig against the NCBI NT database. The fourth column is the relative contribution of this locus tag to this function (e.g. if two genes with equal expression were assigned to one function, both would have 50% contribution to that function). The last four columns show the function of the most similar protein as identified by blastp (based on the locus’ protein sequence) in the Uniprot database, followed by the accession number of the hit, the identity on protein level and the taxonomy of this entry.

Gene(s)	Locus tag	Taxon of closest match	Contribution to function (%)	Best blast hit ^a^	Accession number of the blast hit	Identity (%)	Taxonomy of the best blast hit
*ubiD*	Contig-100_0_8^b^	Unclassified *Clostridia*	31	Putative anaerobic benzene carboxylase *abcA*	D8WWP8	98	BF^c^
*ubiD*	Contig-100_751_1	BF	21	Putative 3-polyprenyl-4-hydroxybenzoate carboxy-lyase	D8WWN4	99	BF
*ubiX*	Contig-100_0_6	Unclassified *Clostridia*	12	Putative UbiX-like carboxylase	D8WWQ0	96	
*bzlA*	Contig-100_0_7	Unclassified *Clostridia*	99	Putative benzoate-CoA ligase BzlA	D8WWP9	96	BF
*ppsA*	Contig-100_29_8	BF	69	Putative phenylphosphate synthase PpsA	D8WWB1	78	BF
*ppsB*	Contig-100_29_7	BF	31	Putative phosphoenolpyruvate synthase/putative phenylphosphate synthase PpsB	D8WWQ5	85	BF
*ppcC*	Contig-100_0_9	Unclassified *Clostridia*	96	Putative anaerobic benzene carboxylase AbcD	D8WWP7	97	BF
*hcrL* ^d^	—	—	—	—	—	—	—
*hcrA*	Contig-100_79_3	BF	100	Putative 4-hydroxybenzoyl-CoA reductase alpha subunit	D8WWW1	95	BF
*bcrA/badF/bzdQ*	Contig-100_418_1	*Candidatus* Kuenenia stuttgartiensis	18	Uncharacterized Protein	Q1Q1I6	98	*Candidatus* Kuenenia stuttgartiensis
*bcrA/badF/bzdQ*	Contig-100_91_3	*Desulfotomaculum gibsoniae*	48	CoA-substrate-specific enzyme active	K8E0C9	73	*Desulfotomaculum hydrothermalte* Lam5
*bamB*	Contig-100_37_6	BF	58	Putative aldehyde ferredoxin oxidoreductase	D8WWJ6	85	BF
*bamC*	Contig-100_37_5	BF	18	Putative benzoate-degrading protein BamC	D8WWR7	82	BF
*bamD*	Contig-100_37_4	BF	11	Putative benzoate-degrading protein BamD	D8WWD0	90	BF
*bamE*	Contig-100_37_2	BF	3	Heterodisulfide reductase subunit A/putative benzoate-degrading protein BamE	A0A101WHV3/D8WWG6	78/80	*Desulfosporosinus* sp. BRH_c37/BF
*bamI*	Contig-100_37_3	BF	7	Sulfur carrier protein FdhD	A0A0A2U5N3	72	*Desulfosporosinus* sp. Tol-M
*bzdW*	Contig-100_24_5	BF	100	Uncharacterized Protein	A0A0F2S5R7	78	*Peptococcaceae* bacterium BRH_c23
*bzdX*	Contig-100_24_4	BF	100	Alcohol dehydrogenase	A0A0J1I9E0	68	*Peptococcaceae* bacterium CEB3
*bzdY*	Contig-100_24_6	BF	100	Putative 6-oxocyclohex-1-ene-1-carbonyl-CoA hydratase BzdY	D8WWK5	93	BF
*pimE*	Contig-100_24_2	BF	70	Putative carboxyl transferase	D8WWL0	91	BF
*pimB*	Contig-100_24_7	BF	67	3-ketoacyl-CoA thiolase	A0A0F2JL78	78	*Desulfosporosinus* sp. I2
*acd*	Contig-100_40_1	*Desulfosporosinus youngiae*	100	Putative acyl-CoA dehydrogenase	D8WWL1	84	BF
*gcdB*	Contig-100_40_5	*Desulfosporosinus youngiae*	100	Sodium ion-translocating decarboxylase, beta subunit	R4KCY5	70	*Desulfotomaculum gibsonia* DSM7213
*gcdC*	Contig-100_40_3	*Desulfosporosinus youngiae*	100	Acetyl/propionyl-CoA carboxylase, alpha subunit	L0HNW4	43	*Aciduliprofundum* sp. strain MAR08–339
*gcdH*	Contig-100_24_1	BF	63	Putative acyl-CoA dehydrogenase	D8WWL1	89	BF
*paaF* ^*e*^	—	—	—	—	—	—	—
*paaH* ^f^	—	—	—	—	—	—	—
*pcaF*	Contig-100_5019_1	Unclassified bacteria	100	Acetyl-CoA acetyltransferase	A0A0M2U9B1	64	*Clostridiales* bacterium PH28_bin88
*tmoA*	contig-100_165_1	Unclassified *Proteobacteria*	41	Methane/phenol/toluene hydroxylase:YHS	N6YH50	96	*Thauera* sp. 27
*tmoA*	contig-100_78_2	*Pseudomonas aeruginosa*	59	Toluene monooxygenase	A0A0C5J946	87	*Rugosibacter aromaticivorans*
*tmoB*	contig-100_165_2	Unclassified *Proteobacteria*	82	Toluene-4-monooxygenase system B	N6XZS7	91	*Thauera* sp. 63
*tmoB*	contig-100_78_3	*Pseudomonas aeruginosa*	18	Toluene monooxygenase	A0A0F2QUZ5	81	*Pseudomonas* sp. BRH_c35
*tmoC*	contig-100_78_7	*Pseudomonas aeruginosa*	100	Oxidoreductase	A0A0F2QUY4	75	*Pseudomonas* sp. BRH_c35
*tmoD*	contig-100_165_4	Unclassified *Proteobacteria*	40	Toluene 4-monooxygenase protein D	Q479D6	66	*Dechloromonas aromatica* strain RCB
*tmoD*	contig-100_78_5	*Pseudomonas aeruginosa*	60	Monooxygenase	A0A0C5J8Z1	71	*Rugosibacter aromaticivorans*
*tmoE*	contig-100_165_5	Unclassified *Proteobacteria*	21	Toluene 4-monooxygenase protein E	Q479D7	91	*Dechloromonas aromatica* strain RCB
*tmoE*	contig-100_78_6	*Pseudomonas aeruginosa*	79	Toluene monooxygenase	A0A0C5J9A6	84	*Rugosibacter aromaticivorans*
*tmoF*	contig-100_165_3	Unclassified *Proteobacteria*	65	Rieske (2Fe-2S) region	N6YA68	87	*Thauera* sp. 27
*tmoF*	contig-100_78_4	*Pseudomonas aeruginosa*	35	Toluene-4-monooxygenase system protein C (Belongs to CMGI-2)	Q1LNS9	73	*Cupriavidus metallidurans* strain ATCC 43123
*dmpK*	contig-100_3910_1	*Pseudomonas aeruginosa*	100	Phenol 2-monooxygenase P0 subunit	Q479F5	92	*Dechloromonas aromatica* strain RCB
*dmpL*	contig-100_2025_1	Unclassified *Rhodocyclaceae*	51	Phenol 2-monooxygenase P1 subunit	Q479F6	77	*Dechloromonas aromatica* strain RCB
*dmpL*	contig-100_3910_2	Dechloromonas aromatica	49	Phenol 2-monooxygenase P1 subunit	Q479F6	98	*Dechloromonas aromatica* strain RCB
*dmpM*	contig-100_2025_2	Unclassified *Rhodocyclaceae*	100	Phenol 2-monooxygenase P2 subunit	Q479F7	97	*Dechloromonas aromatica* strain RCB
*dmpN*	contig-100_1081_2	Unclassified *Rhodocyclaceae*	88	Phenol 2-monooxygenase P3 subunit	Q479F8	84	*Dechloromonas aromatica* strain RCB
*dmpO*	contig-100_1081_1	Unclassified *Rhodocyclaceae*	89	Phenol 2-monooxygenase P4 subunit	Q479F9	78	*Dechloromonas aromatica* strain RCB
*dmpP*	contig-100_2834_2	Azoarcus toluclasticus{92003}	100	Phenol 2-monooxygenase	N6YI82	79	*Thauera* sp. 63
*dmpB*	contig-100_1413_1	*Candidatus* Kuenenia stuttgartiensis	71	Similar to cysteine dioxygenase type I	Q1PVP4	94	*Candidatus* Kuenenia stuttgartiensis
*dmpC*	contig-100_1829_1	*Candidatus* Kuenenia stuttgartiensis	73	Similar to succinate-semialdehyde dehydrogenase [NADP + ]	Q1Q6T5	91	*Candidatus* Kuenenia stuttgartiensis
*dmpD*	contig-100_761_1	N/A	100	2-hydroxymuconate semialdehyde hydrolase	Q479G6	89	*Dechloromonas aromatica* strain RCB
*dmpE*	contig-100_761_2	N/A	100	Hydratase/decarboxylase	Q479G7	84	*Dechloromonas aromatica* strain RCB
*dmpF*	contig-100_4851_2	*Azoarcus* sp. BH72	74	Acetaldehyde dehydrogenase	A0A0K1JCI5	92	*Azoarcus* sp. CIB
*dmpG*	contig-100_6348_1	*Limnobacter* sp. MED105	69	4-hydroxy-2-oxovalerate aldolase	A0A0K1JC70	89	*Azoarcus* sp. CIB
*dmpH*	contig-100_1640_1	*Thermincola potens*	39	2-keto-4-pentenoate hydratase/2-oxohepta-3-ene-1,7-dioic acid hydratase	K6T593	71	*Methanobacterium* sp. Maddingley MBC34
*dmpH*	contig-100_3980_1	Unclassified bacteria	42	2-hydroxyhepta-2,4-diene-1,7-dioate isomerase	A0A0P6XMJ4	69	*Ornatilinea apprima*
*dmpI*	contig-100_834_2	*Desulfosporosinus orientis*	71	Tautomerase	A0A101WBV1	72	*Desulfosporosinus* sp. BRH_c37

^a^Based on uniprot May 11, 2016.

^b^Contig-100 is the default IDBA_UD output for a kmer-run of 100, the following number is the contig number and last number is the gene number on that contig.

^c^*Clostridia* bacterium enrichment culture clone BF.

^d^All potential assignments overlap with *bzlA*.

^e^All potential assignments overlap with *pimE*.

### Benzene activation mechanisms

We did not find transcripts indicating methylation of benzene to toluene (the proposed pathway is shown in Figure S1). The *bssA* gene encoding the α-subunit of the key enzyme benzylsuccinate synthase was also not detected by qPCR in the co-extracted DNA samples^40^. In line with our results, genes of the toluene activation pathway were absent in a metatranscriptomic study conducted using another benzene-degrading nitrate-reducing culture^42^, although benzene methylation mechanism was proposed for this culture in the past^16^. To date, known benzene-degrading anaerobes do not seem to employ activation by methylation as (i) no proteins mediating benzene methylation were found in a proteogenomic analysis of a benzene-degrading culture that used iron as the electron acceptor^41^, (ii) none of the investigated benzene-degrading pure cultures seems to employ a methylation step for benzene activation, (iii) no intermediates such as the key product of anaerobic toluene activation, benzylsuccinate, have been detected, and (iv) some anaerobic benzene-degrading enrichment cultures failed to degrade toluene^12,15,35^.

We found transcripts potentially involved in benzene hydroxylation to phenol (Fig. 2A,C). Among these was a polycistronic transcript that contained genes for the synthesis of UbiD and UbiX, along with a hydroxylase candidate (contig-100_193). The hydroxylase candidate showed low identity (58% at the amino acid level) to a NUDIX family hydrolase from the deltaproteobacterial strain NaphS2^46^ that is not reported to be involved in anaerobic benzene activation. In addition, we found transcripts of phenylphosphate synthase genes (*ppsABC*) and phenylphosphate carboxylase genes (*ppcC*). We also detected transcripts of a gene similar to the *hcrL* gene encoding 4-hydroxybenzoate-CoA ligase, however, it is not possible to differentiate between *hcrL* and the benzoate-CoA ligase gene (*bzlA*) (Table 1). Generally, the specificity of the CoA ligases for 4-hydroxybenzoate and benzoate is difficult to predict solely on the basis of sequence similarity^47,48^. The transcript of a 4-hydroxybenzoyl-CoA reductase gene (*hcrA*) was also identified. Taken together, these findings might indicate hydroxylation of benzene to phenol in this consortium. Anaerobic benzene oxidation via phenol was documented for *G*. *metallireducens*^18,19^. However, besides lack of an identifiable hydroxylase, we did not find a full set of transcripts encoding all subunits of the essential enzymes for this pathway in our study. Likewise, the reconstructed genome of the *Pelotomaculum* candidate BPL did not show a full repertoire of genes involved in anaerobic phenol degradation^49^.

The high level of transcripts involved in the proposed anaerobic benzene carboxylase pathway (*abcA* in contiq_100_0_8 and contig-100_751_1, and *abcD* in contiq_100_0_9) and a benzoate-CoA ligase gene (*bzlA* contiq_100_0_7)^41^ as revealed in this study (Fig. 2A,C, Table 1) corroborates that benzene carboxylation to benzoate is the main initial benzene degradation pathway in our culture. In line with our results, genes encoding UbiD/UbiX-related carboxylases were also highly transcribed in yet another benzene-degrading nitrate-reducing enrichment, suggesting benzene carboxylation to benzoate as the main mechanism of anaerobic benzene activation^42^. Similarly, the hyperthermophilic archaeon *F*. *placidus* was proposed to employ a benzene-induced UbiD-related benzene carboxylase (Frep_1630) for anaerobic benzene oxidation^17^. Although biochemical data to demonstrate benzene carboxylation is pending, the compiling evidence on carboxylation of benzene^17,41,42^ and naphthalene^46,50–52^ indicates carboxylation as an important initial reaction involved in the anaerobic degradation of non-substituted aromatic hydrocarbons^7,53^. Most recently, a novel UbiD-related decarboxylase was shown to mediate anaerobic phthalate degradation by decarboxylation of phthaloyl-CoA to benzoyl-CoA, further reinforcing the importance of UbiD-related (de)carboxylases in anaerobic degradation of aromatic compounds^54^.

### Co-localization of the putative genes involved in benzene carboxylation

The putative benzene carboxylation genes transcribed in this study showed high similarity (>96% at the amino acid level) and gene synteny to a cluster previously proposed to encode putative enzymes for benzene carboxylation to benzoate^41^ (Figure S2). Similar observations were made with another benzene-degrading nitrate-reducing enrichment culture indicating a highly conserved set of genes involved in benzene carboxylation in these types of enrichments^42^. Noteworthy, the three enrichments in which these gene clusters were identified were obtained from geographically distinct locations in Poland^32,41^, Canada^37,42^ and the Netherlands^38,40^, and operated under iron-reducing^32,41^ or nitrate-reducing conditions^37,38,40,42^. Similarly, gene clusters encoding enzymes involved in carboxylation reactions in the anaerobic degradation of naphthalene are co-localized in the genomes of the sulfate-reducing cultures N47^52^ and NaphS2^46^. The genes for the degradation of aromatic compounds are usually clustered at a single genomic locus^55^. Furthermore, the co-localization and co-transcription of genes encoding a transcriptional regulator, MarR, and multidrug resistance protein MRP homologue (Figure S2) suggest a functional relationship between these genes and the *abcAD* and *bzlA* genes. As such, in the genome of the facultatively anaerobic benzoate-degrading *Thauera aromatica* and the phototrophic bacterium *Rhodopseudomonas palustris*, *marR* is co-localized with benzoate degradation genes and proposed to regulate their transcription^56–58^. In contrast, the proposed gene encoding a UbiD-related carboxylase in *F*. *placidus* (Frep_1630) is not co-localized with genes coding for carboxylase proteins, benzoate-CoA ligase proteins, or any other proteins involved in the metabolism of aromatic compounds^17^, even though most of the other genes involved in anaerobic aromatic degradation in *F*. *placidus* are localized within gene clusters^17,59^. Genes homologous to *abcA* were also present in the genome of *Pelotomaculum* candidate BPL (single copy with 33% amino acid sequence identity)^49^ and in the metagenomes of hydrocarbon-degrading enrichment cultures^60,61^. However, genes homologous to *abcD*^49,60,61^ or *bzlA*^49,60^ were absent.

Another interesting finding in our study was transcripts of genes for phage-related proteins and transposable elements some of which were located within the same contig that contained putative aromatic-degrading genes (e.g. contig-100_0; Table S3). This implies potential distribution of xenobiotic degradation genes by horizontal gene transfer^62^.

### Upper pathway

#### Dearomatization

Reductive dearomatization of the benzene ring by benzoyl-CoA reductase (BCR) is the key step in anaerobic degradation of benzoyl-CoA, and BCR is the only oxygen-sensitive enzyme within the benzoyl-CoA pathway^55^. There are two types of BCRs: class I are ATP-dependent FeS enzymes composed of four different subunits^63^ whereas class II are ATP-independent enzymes that contain eight subunits and harbour a tungsten-containing cofactor in the active site^64^. All known monoaromatic-degrading strict anaerobes apply class II BCRs with the exception of the benzene-degrading archaeon *F*. *placidus* that lacks the genes coding for the class II BCRs^59^ and employs an ATP-dependent *Azoarcus*-type BCR^65^. We found transcription of *bam*-like genes (*bamBCDEI*, from strict anaerobes^55^) and at much lower relative abundance genes analogous to one subunit of class I BRC (*bzdQ* and its homologs *bcrA*/*badF*, from facultative anaerobes^55^) in our enrichment culture (Fig. 2A,C, Table 1). This finding indicates that class II BCRs are recruited similar to strictly anaerobic microorganisms. In accordance with our results, Bam-like proteins were detected in a proteogenomic analysis of a benzene-degrading and iron-reducing enrichment culture, indicating that benzoyl-CoA reduction steps are analogous to the activities of class II BCRs^41^. Genomic and proteomic evidence also proposed benzoate-CoA degradation via Bam-like BCR by *Pelotomaculum* candidate BPL^49^. Similarly, *bam*-like genes were almost exclusively transcribed in a nitrate-reducing enrichment culture growing on benzene but not when it was growing on benzoate^42^.

#### Modified β-oxidation

Modified β-oxidation of the dearomatized diene product (cyclohexadienoyl-CoA) by specific hydratases, dehydrogenases and hydrolases results in ring cleavage and diene conversion to an aliphatic C7-dicarboxyl-CoA (Fig. 2A,C). The β-oxidation reactions are similar in facultative and strict anaerobes^55^. We found transcription of *Azoarcus*-type *bzdXYW* genes^66^ (Fig. 2A,C, Table 1) indicating that the modified β-oxidation reactions in our culture are related to those of denitrifying bacteria. The *bzd* genes are located in a catabolic operon (*bzdNOPQMSTUVWXYZA*) in *Azoarcus* sp. strain CIB^66^. The *bzdXYW* gene transcripts identified in our dataset were similarly co-located (contig100_24_4 to contig100_24_6, Table 1) implying a functional relationship. Transcripts of *bzdXYW*-like genes from *Azoarcus* were also identified in two other benzene-degrading enrichment cultures^41,42^.

### Lower pathway

The C7-dicarboxyl-CoA is degraded to three acetyl-CoAs and CO_2_ through a series of reactions that involve a dicarboxylic acid β-oxidation pathway (leading to glutaryl-CoA), a glutaryl-CoA dehydrogenase (leading to crotonyl-CoA), and a short-chain fatty acid β-oxidation pathway (leading to two acetyl-CoAs) (Fig. 2A,C)^55^. We found transcription of the *pimE* and *pimB* genes encoding 3-hydroxypimeloyl-CoA dehydrogenase and acetyl-CoA acyltransferase, respectively (Fig. 2, Table 1). These enzymes which link pimeloyl-CoA to the central metabolism via glutaryl-CoA, are best described for *R*. *palustris*, in which they are encoded by the *pim* operon^67^. The subsequent decarboxylation of glutaryl-CoA to crotonyl-CoA is the second reaction in the benzoyl-CoA degradation pathway (the first being the dearomatization of benzoyl-CoA, see above), catalyzed by different enzymes in obligate and facultative anaerobes^7,68^. Facultative anaerobes employ a decarboxylating glutaryl-CoA dehydrogenase with crotonyl-CoA as the product^67^. Obligate anaerobes on the other hand employ a non-decarboxylating glutaryl-CoA dehydrogenase (that forms glutaconyl-CoA as an intermediate) in combination with a glutaconyl-CoA decarboxylase. The latter is sodium-dependent and will allow ATP synthesis by coupling the subsequent decarboxylation of its product (glutaconyl-CoA) with a translocation of sodium ions across the membrane^69^. We found transcription of a non-decarboxylating glutaryl-CoA dehydrogenase encoding gene (*acd*) accompanied by genes that code for a sodium-translocating glutaconyl-CoA decarboxylase (*gcdBC*) on the same contig (contig-100_40) (Fig. 2A,C, Table 1). This implies that energy-conserving mechanisms were employed by our culture, similar to strict anaerobes degrading aromatic compounds e.g. *Syntrophus aciditrophicus*^70^ and *Desulfococcus multivorans*^68^. We also found transcription of a decarboxylating glutaryl-CoA dehydrogenase gene (*gcdH*) in our enrichment culture (Fig. 2A,C, Table 1). However, the assembled transcripts observed here only encoded a rather short fragment of 66 amino acids compared to a usual decarboxylating glutaryl-CoA dehydrogenase of around 400 amino acids in length. Hence, the actual function could not be unambiguously predicted due to the truncated nature of the assembly.

### Transcription of genes involved in aerobic benzene degradation

A striking finding was the identification of transcripts from a full set of genes involved in aerobic benzene degradation (Fig. 2B,C, Table 1). Both toluene monooxygenase (*tmoABCDEF*)^71^ and phenol hydroxylase (*dmoKLMNOP*)^21^ were shown to oxidise benzene to catechol. The catechol 2,3-dioxygenase encoded by *dmpB* mediates oxidative ring cleavage of catechol, which is then further converted to pyruvate and acetyl-CoA by enzymes of the lower pathway encoded by *dmpCDEFGHI*^72^. The *dmp* genes were characterized from the phenol-catabolizing plasmid pVI150 of *Pseudomonas* sp. CF600^72^ and are homologous to *phe* genes from the phenol-utilizing strain *Bacillus thermoglucosidasius* A7^73^, *tdn* genes from the aniline-catabolizing plasmid pTDN1 of *P*. *putida* UCC22^74^, *nah* genes from the naphthalene-catabolizing plasmid NAH7 of *P*. *putida* G7^75^ and *nag* genes from the naphthalene-utilizing strain *Ralstonia* sp. U2^76^.

### Oxygen production in the anaerobic benzene degrading culture

Possible explanations for the observation of transcripts for enzymes involved in aerobic metabolism under nitrate-reducing conditions might be oxygen influx or production in the enrichment culture. It has been shown that oxygen can be produced by a selected set of species that employ a nitric oxide dismutase (Nod) during nitrate reduction^22^. The resulting low concentrations of oxygen can be effectively scavenged in biofilms by the activities of monooxygenases and respiratory enzymes, such that strict anaerobes are protected from oxygen toxicity^77^. As such, biofilms can provide the necessary barrier for spatial separation of anaerobic and aerobic microbes.

To test the possibility of internal oxygen production, we added 0.5 mM nitrite to the continuous culture, but no oxygen production was detected within 2.5 hours. However, after addition of 1 mM formate to stimulate nitrite reduction, an oxygen concentration of up to 2.1% (5.25 µM) was detected by the oxygen electrode in the liquid phase of the continuous culture after 1.5 hours and by headspace oxygen analysis using GC-TCD (Figure S3). Nitrite was depleted after 12 days, and subsequently a second spike of 0.5 mM nitrite and 1 mM formate was added to the continuous culture. This time, no oxygen was detected (oxygen detection limit <0.1%, 0.25 µM). It is tempting to speculate that the aerobic organisms enriched during the first nitrite/formate spike effectively scavenged the oxygen formed during the second spike.

Typical Nod have a tandem histidine, one to ligate the low spin haem, the other to ligate the high spin reaction center haem^78^. This second histidine is absent from the Nod sequences, and therefore a characteristic discriminator between nitric oxide reductases and dismutases^78^. A search for the conserved Nod motifs did not reveal any matches in our dataset, however, this does not rule out an intermediate role for oxygen in the activation of benzene during denitrification. For example, *D*. *aromatica* strain RCB lacks genes encoding enzymes for anaerobic aromatic degradation and for the key enzyme Nod^28^, yet it was reported to degrade benzene under denitrifying conditions^26^. Moreover, the anaerobic methanotroph *Candidatus* Methylomirabilis oxyfera contains the entire pathway for aerobic methane oxidation but lacks key genes for anaerobic methane and hydrocarbon degradation, and activates methane in the presence of nitrite with oxygen and nitrogen formation^22^. Likewise, the alkane-degrading facultative denitrifying γ-proteobacterium strain HdN1 lacks genes for anaerobic alkane degradation but contains genes encoding monooxygenases^25^. However, in contrast to *D*. *aromatica* strain RCB, both *Candidatus* M. oxyfera and γ-proteobacterium strain HdN1 contain highly identical putative Nod^78^. These findings suggest a yet unknown pathway for oxygen formation from nitrate/nitrite that can be used for aerobic hydrocarbon degradation under anoxic conditions.

Transcripts for oxygenases associated with oxidation of monoaromatic compounds, particularly genes of benzoyl-CoA oxygenases (*box* genes), were also reported during growth on benzene and benzoate in a nitrate-reducing enrichment culture^42^. The *box* genes expressed under anoxic conditions in benzoate-degrading *Azoarcus* cultures were proposed to constitute an alternative oxygen-scavenging mechanism^79^ and may assist in a strategy to rapidly shift to aerobic degradation if oxygen levels become higher^4,79^.

Compound specific isotope analysis (CSIA) might help to further elucidate benzene biodegradation mechanisms. Interestingly, a recent combined carbon (C) and hydrogen (H) CSIA showed that isotope enrichment in a benzene-degrading nitrate-reducing enrichment culture (Λ^C/H^ = 12 ± 3)^80^ was distinct from the same culture grown under sulfate-reducing condition (Λ^C/H^ = 28 ± 3)^81^. In turn, it was similar to the isotope fractionation patterns of aerobic benzene degraders employing monooxygenase i.e. *Cupriavidus necator* ATCC 17697 (Λ^C/H^ = 11 ± 6) and *Alicycliphilus denitrificans* strain BC (Λ^C/H^ = 10 ± 4)^81^. This suggests involvement of monooxygenase-mediated degradation under nitrate-reducing condition^80^. Unfortunately, we were unable to grow our continuous culture in batch cultures for CSIA even when biofilm material was used as inoculum.

### Transcription of genes involved in nitrate metabolism

We found transcripts from a number of genes involved in nitrate reduction (*narGHI/nrxAB*, *nirK*, *norB*, *nosZ*, *nrfAH)*, including both denitrification and dissimilatory nitrate reduction to ammonium (DNRA). Interestingly, among the genes necessary for stepwise denitrification, transcription of the *narGHI* and *nirK* genes (mediating reduction of nitrate - > nitrite - > nitric oxide) was higher than that of the downstream *norB* and *nosZ* genes (mediating reduction of nitric oxide - > nitrous oxide - > dinitrogen) (Figure S4, Table S4). This suggests that nitrous oxide is not likely the main product of nitric oxide reduction. We also identified transcripts for assimilatory nitrate reduction (*nsaA*, *narB*), nitrogen fixation (*nifDH*) and nitrification (*amoAB*) (Figure S4, Table S4). The latter might also indicate oxygen presence in the culture.

In summary, our metatranscriptomic study of a benzene-degrading nitrate-reducing continuous culture provides insights into benzene degradation mechanisms. This culture appears to activate benzene predominantly via carboxylation, and employs ATP-independent BCR similar to what has been reported for strict anaerobes. The downstream pathway resembles that found in facultative anaerobes except for a non-decarboxylating glutaryl-CoA dehydrogenase that might enable energy conservation similar to strict anaerobes.

The likelihood of oxygen production from nitrate reduction proposed in our study and elsewhere^80^ is in agreement with field data. For example, a recent study showed unexpected diversity and high abundance of putative *nod* genes in BTEX-contaminated aquifers^82^. Interestingly, ample *nod* sequences were retrieved from the highly reduced core of an anoxic BTEX plume^82^ for which high abundance of *tmoA* genes had previously been revealed^83^. Likewise, a metagenomic study of anoxic hydrocarbon resource environments that had been subjected to nitrate injection showed high proportions of genes for enzymes involved in aerobic hydrocarbon metabolism^84^. Oxygenic denitrifiers may offer ecological advantages by enabling the aerobic microbes to thrive in hydrocarbon-contaminated anoxic subsurface environments.

## Methods

### Enrichment culture

A chemostat (Applikon, Schiedam, the Netherlands) culture that originated from soil samples obtained from a benzene-contaminated site located in the northern part of the Netherlands has been maintained with benzene as electron donor and nitrate as electron acceptor for more than 15 years^38^. Details of media composition and culture conditions were described previously^40^.

### Sampling, RNA extraction and sequencing

Biofilms grown on the glass wall of the reactor had different morphologies^40^. Four suspended biofilm samples were taken from the areas with white biofilm: three on 31^st^ October 2014 and one on 3^rd^ November 2014. Moreover, two suspended biofilm samples were taken from the areas with brown biofilm on 3^rd^ November 2014 (Table S1). Defined areas of biofilm attached to the glass wall were scraped off under a constant N_2_/CO_2_ (80/20%) flow. The liquid phase in the vessel was stirred for 5 minutes at 200 rpm to dislodge the biofilm aggregates followed by liquid phase sampling as described previously^40^. The samples were immediately stored at −80 °C. DNA and RNA co-extraction and purification was done as described previously^85^. The DNA samples were used for community analysis using MiSeq sequencing and quantification of key benzene degradation genes as described elsewhere^40^. The RNA samples were used for metatranscriptomic analysis in this study. Removal of rRNA, synthesis of cDNA and adding indices for Illumina library preperation were performed using the ScriptSeq^TM^ Complete Kit (Bacteria) (Epicentre) following the manufacturer’s protocol. Single read sequencing was done with an Illumina HiSeq. 2500 (GATC-Biotech, Konstanz, Germany) generating reads between 6.02 to 46.4 M per sample. The read length was 150 bp.

### Data quality assessment and filtering

SortMeRNA v1.9^86^ was used to remove rRNA reads. Trueseq adapters were trimmed with cutadapt v1.2.1^87^ with the –b settings. Quality trimming was performed with PRINSEQ Lite v0.20.2^88^, with a minimum sequence length of 40 bp and a minimum quality of 30 on both ends of the read and as mean quality. All reads with non-IUPAC characters were discarded as well as reads containing more than three Ns.

### Assembly and annotation

All reads which passed the quality assessment were pooled and cross-assembled with IDBA_UD version 1.1.1 with standard parameters^89^. All contigs, which contained more than 90% of a single base, more than 90% GC or AT, or which contained 50 or more bases of the same type in a row, were removed from further processing. On the assembled meta-transcriptome, Prodigal v2.5 was used for prediction of protein coding DNA sequences (CDS) with the option for meta samples^90^. Reads were mapped to the meta-transcriptome with Bowtie2 v2.0.6^91^ using default settings. BAM files were converted with SAMtools v0.1.18^92^, and gene coverage was calculated with subread version 1.4.6^93^.

The proteins were annotated with KAAS^94^, with SBH and ghostX as settings and with InterProScan 5.6-48.0^95^. The annotation was further enhanced by adding EC numbers via PRIAM version March 06, 2013^96^. Further EC numbers were derived by text mining and matching all InterproScan derived domain names against the BRENDA database (download 13.06.13)^97^. This text mining was done as outlined in supporting information. All EC and KO numbers were mapped with custom scripts onto the KEGG database^98^ and visualized using Python Scipy version 1.6.1 and NumPy version 0.9.0^99^.

### Taxonomic assignments

All assembled contigs were analysed with Blast 2.2.29^100^ against the NCBI NT database (download 22.01.2014) with standard parameters besides an e-value of 0.0001, the human microbiome (download 08.05.2014), the NCBI bacterial draft genomes (download 23.01.2014), the NCBI protozoa genomes (download 08.05.2014), and the human genome (download 30.12.2013, release 08.08.2013, NCBI Homo sapiens annotation release 105). Taxonomy was estimated with a custom version of the LCA algorithm as implemented in MEGAN^101^, but with changed default parameters. Only hits exceeding a bitscore of 50 were considered and of these only hits with a length of more than 100 nucleotides and that did not deviate more than 10% from the longest hit were used. All contigs, for which this did not result in any assignment, were again analysed with Blast using all the above mentioned databases, but with the –blastn option and the taxonomic assignment was calculated as mentioned.

### Testing oxygen production

The oxygen production experiment was performed at a dilution rate of 0.1 day^−1^ at 25 °C with an influent benzene concentration of 100 µM as previously described^40^. The influent medium was similar to the medium used for metatranscriptomic analyses except that the vitamins were excluded and (NH_4_)_2_SO_4_ was replaced with 1.9 mM Na_2_SO4. The oxygen production was measured using an oxygen electrode submerged in the liquid phase of the continuous culture (AppliSens, Applikon). The oxygen electrode was calibrated by sparging nitrogen gas (0% O_2_) or air (100% O_2_) through demineralized water at 20 °C corresponding to 0 µM or 250 µM dissolved oxygen, respectively. The oxygen detection limit was 0.1% (0.25 µM). Headspace oxygen concentrations were measured with a Varian 3800 gas chromatographic (GC) system equipped with a thermal conductivity detector (TCD) and a tandem column (Molsieve 5 A/Porabond Q, Agilent, CA, USA). The TCD detector was set at 80 °C and the filament temperature was 160 °C. The oven temperature was constant at 45 °C for 8 min with helium as carrier gas. Headspace samples of 250 µl were taken from the continuous culture with a 250 µl Pressure-Lock gas syringe and a 0.6 × 25 mm sterile needle (Henke Sass Wolf, Tuttlingen, Germany) followed by 50 µl injection into the GC-TCD. Nitrite was added from a 1 M anoxic stock solution (NaNO_2_) to a final concentration of 0.5 mM. Formate was added from a 2 M anoxic stock solution (HCOONa) to a final concentration of 1 mM. Benzene was measured in 0.5 ml headspace samples of the reactor on a GC-FID system, as described previously^40^.

### Sequence Data

All sequence data from this study were deposited at the European Bioinformatics Institute under the accession numbers ERS1670018 to ERS1670023. Further, all assigned genes, taxonomy, function, sequences of contigs, genes and proteins can be found in Table S3.

## Electronic supplementary material

Supplementary information

Dataset1
