# Supplementary material for: A benzene-degrading nitrate-reducing microbial consortium displays aerobic and anaerobic benzene degradation pathways
Source: Sci Rep. 2018 Mar 14;8:4490. doi: 10.1038/s41598-018-22617-x (PMC5852087; doi:10.1038/s41598-018-22617-x)
Supplement: Supplementary file 1 — Supplementary information [file 41598_2018_22617_MOESM1_ESM.pdf]

Supporting information

A benzene-degrading nitrate-reducing microbial consortium displays aerobic and anaerobic benzene degradation pathways

Siavash Atashgahi,[1*#] Bastian Hornung,[2#] Marcelle J van der Waals,[1,3] Ulisses Nunes da Rocha,[4,5] Floor Hugenholtz,[1] Bart Nijsse,[2] Douwe Molenaar,[4] Rob van Spanning,[4] Alfons J.M. Stams,[1,6] Jan Gerritse[3] & Hauke Smidt[1]

[1] Wageningen University & Research, Laboratory of Microbiology, Stippeneng 4, 6708 WE Wageningen, the Netherlands
[2] Wageningen University & Research, Laboratory of Systems and Synthetic Biology, Stippeneng 4, 6708 WE Wageningen, the Netherlands
[3] Deltares, Subsurface and Groundwater Systems, Daltonlaan 600, 3584 BK Utrecht, the Netherlands
[4] Vrije Universiteit Amsterdam, Department of Molecular Cell Biology, De Boelelaan 1108, 1081 HZ Amsterdam, the Netherlands
[5] Department of Environmental Microbiology, Helmholtz Centre for Environmental Research - UFZ, Leipzig, Germany
[6] Centre of Biological Engineering, University of Minho, Braga, Portugal

[*] For correspondence: E-mail: siavash.atashgahi@wur.nl, Tel: +31 317 484683

[#] Equal contribution

Text mining

All domains derived from InterproScan were matched against the Brenda database with the following procedure. The text mining algorithm included lower casing all characters, removal of non-alphanumerical characters (colons, commas, brackets, apostrophes, dashes, terminal points), removal of partial and generic terms (type, terminal, subunit, domain, enzyme, like, hypothetical, conserved, operon, active site, probably, central, 51 kd, respiratory chain, c terminal, n terminal), manual rejection of too generic final result terms (kinase, cytochrome, protein, methyltransferase) and reduction of certain terms (deletion of PEP/pyruvate binding, removal of "prokaryotic" in "prokaryotic cytidylate kinase"; "family" in "cytidilate kinase family"; "phosphorylating" in "glyceraldehyde phosphate dehydrogenase phosphorylating"; "iron containing" in "iron containing alcohol dehydrogenase"; "zinc containing" in "zinc containing alcohol dehydrogenase"; "manganese containing" in "manganese containing catalase"; "20 kd" in "nadh ubiquinone oxidoreductase 20 kd", replacement of "carboxyltransferase" with "carboxylase" in "pyruvate carboxyltransferase"). Furthermore all terms containing only one character were also removed in case the remaining name contained more than two words.

Figure S1. Proposed anaerobic biodegradation of benzene via methylation to toluene mediated by an unknown presumed methylase (shown by a question mark) followed by fumarate addition to form benzylsuccinate that is subsequently metabolized to benzoyl-CoA. The genes encoding different enzymes of this pathway are: *bssABC*, benzylsuccinate synthase; *bbsEF*, succinyl-CoA:(R)-benzylsuccinate CoA-transferase; *bbsG* (R)-benzylsuccinyl-CoA dehydrogenase; *bbsH*, phenylitaconyl-CoA hydratase; *bbsCD*, 2-[hydroxy(phenyl)methyl]-succinyl-CoA dehydrogenase; *bbsAB,* benzoylsuccinyl-CoA thiolase.

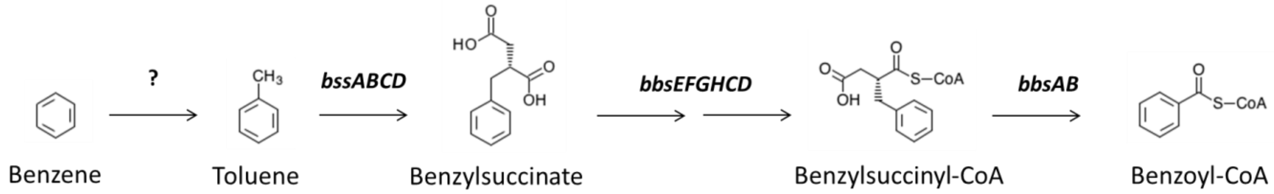

Figure S2. Putative benzene degradation gene cluster of the iron-reducing culture BF [1] (A) and the nitrate-reducing culture used in this study (B) where Contig-100 is the default IDBA_UD output for a kmer-run of 100, the following number is the contig number (in this case 0) and the last number is the gene number on that contig (shown above each corresponding gene).

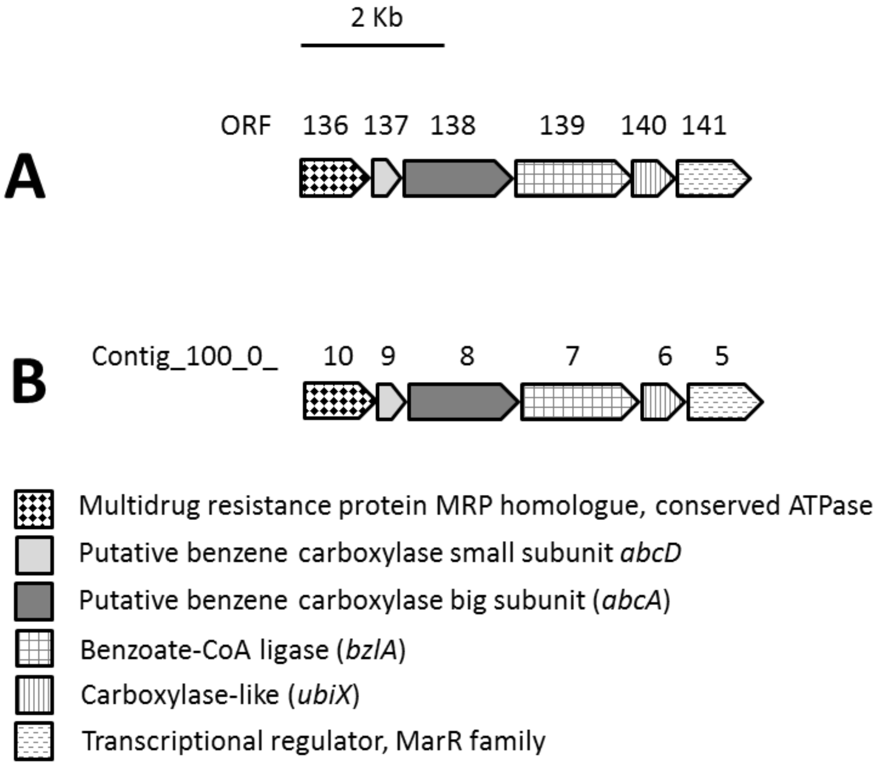

Figure S3. Oxygen concentration in the continuous culture. The black line with circles indicates the oxygen concentration (%) in the culture liquid, measured with an oxygen electrode. The red diamonds indicate oxygen in the headspace gas, measured with a GC-TCD. A spike of 0.5 mM nitrate to the continuous culture is indicated by arrow 1. After 2.5 hours of constant oxygen levels, a spike of 1 mM formate (arrow 2) was added, after which the oxygen concentration increased to 2.2% within 1.5 hours.

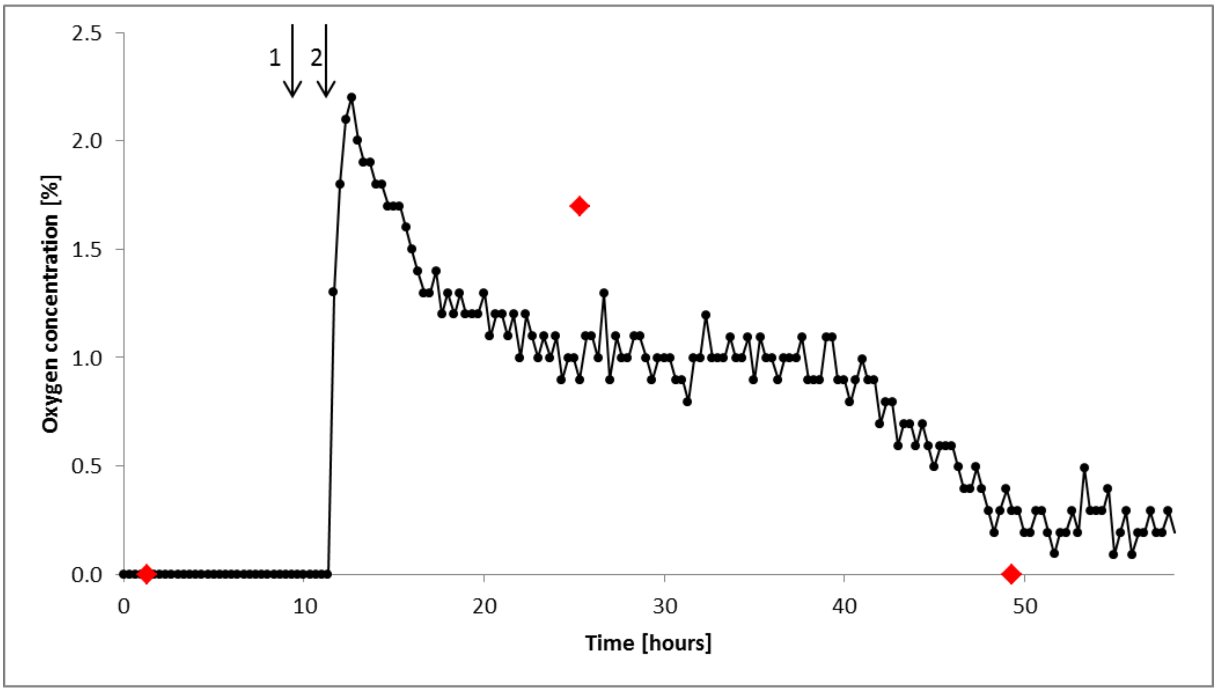

Figure S4. Relative abundances (%) of gene transcripts identified in biofilm samples involved in nitrate metabolism. The bar showing the number of relative abundance was log scaled and 0 values were removed.

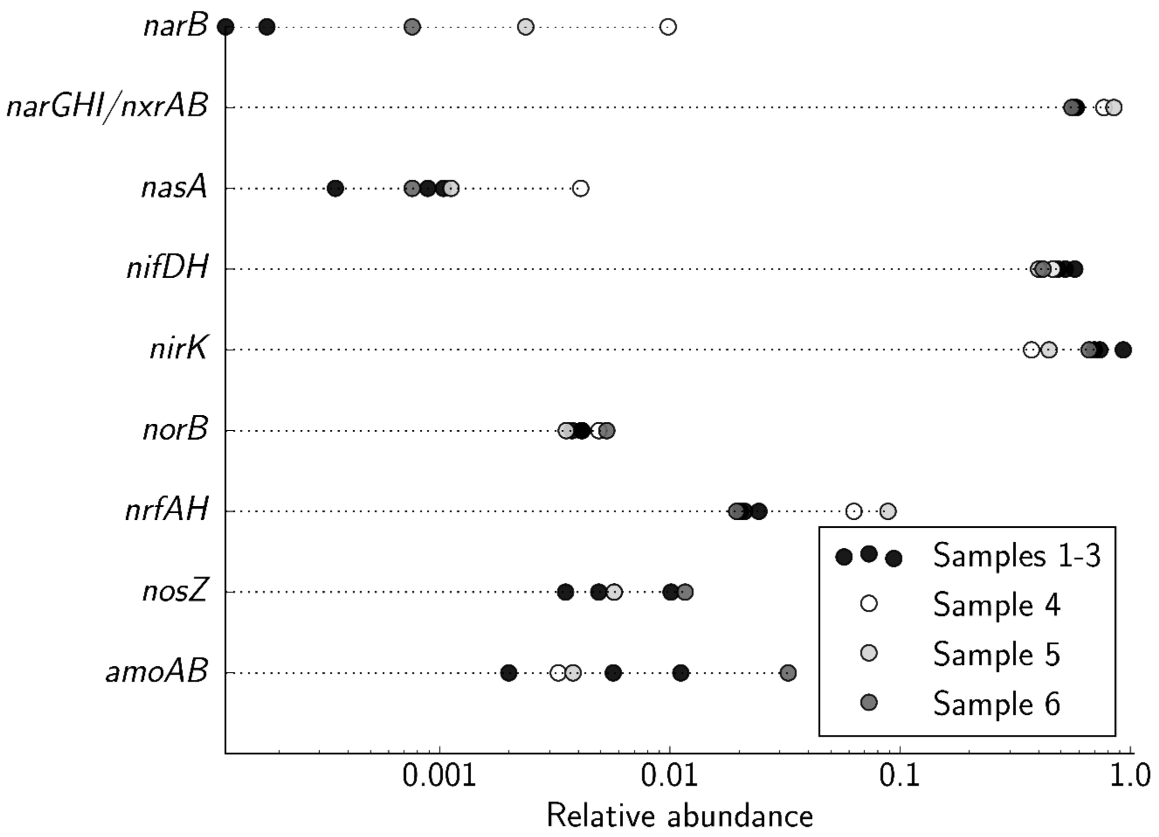

Table S1. Details of samples used in this study. The total amount of biofilm protein in the culture vessel was assessed by extrapolating the amount of protein released from defined surface areas to the total area of biofilm covered surfaces in the system. Protein content in the liquid (15 mg), white (233 mg) and brown biofilm (2485 mg) was used to calculate the protein content percentage in the liquid and biofilm in the samples. Protein content was analyzed as described [2].

**#**	**Biofilm morphologies**	**Sampling date**	**Samples volume (ml)**	**Scraped off biofilm area (cm^2)**		**Protein content (%)**	
				**White biofilm**	**Brown biofilm**	**Liquid**	**Biofilm**
Sample 1	White	31-10-2014	50	6.3	0	89	11
Sample 2	White	31-10-2014	50	6.3	0	89	11
Sample 3	White	31-10-2014	50	6.3	0	89	11
Sample 4	White	3-11-2014	50	6.3	0	89	11
Sample 5	Brown	3-11-2014	50	22.3	4	30	70
Sample 6	Brown	3-11-2014	50	22.3	8	0	100

Table S2. Sequence information summary for the bioreactor biofilm and effluent samples during growth on benzene and nitrate.

Samples	Read number	Non-rRNA read (%)	Reads that passed PRINSEQ Lite quality filtering (%)	Read mean length (bp)	Percentage of reads passing all quality control	Mapping rate of reads passing quality control to the assembly
Sample 1	46403410	46282920 (99.7)	977319 (2.1)	97	2.1	54.6
Sample 2	6768045	6746142 (99.7)	367039 (5.4)	93.22	5.4	53.7
Sample 3	12225282	11938239 (97.7)	3580969 (30)	97.66	30.0	68.3
Sample 4	1169416	1089029 (93.1)	565017 (51.9)	126.37	51.9	78.3
Sample 5	6015250	5694797 (94.7)	2267743 (39.8)	127.87	39.8	84.7
Sample 6	11080970	10896851 (98.3)	1175823 (10.8)	126.33	10.8	81.9

Table S4. Summary of transcribed genes involved in nitrogen metabolism. The first column lists the transcribed genes followed by the locus tag of each gene. The third column shows the taxonomy of the locus tag, based on megablast/blastn hits of the whole contig against the NCBI NT database. The fourth column is the relative contribution of this locus tag to this function (e.g. if two genes with equal expression were assigned to one function, both would have 50% contribution to that function). The last four columns show the function of the most similar protein as identified by blastp (based on the locus' protein sequence) in the Uniprot database, followed by the accession number of the hit, the identity on protein level and the taxonomy of this entry, respectively.

Gene(s)	Locus tag	Taxon of closest match	Contribution to function (%)	Best blast hit [a]	Accession number of the blast hit	Identity (%)	Taxonomy of the best blast hit
narB	Contig-100_6458_1 [b]	*Candidatus* Kuenenia stuttgartiensis	60	Similar to FeS molybdopterin oxidorecutcase proteins	Q1PVH1	94	*Candidatus* Kuenenia stuttgartiensis
narG/nxrA	Contig-100_26_5	unclassified_Bacteria	37	Uncharacterized protein	A0A0B5GVF0	43	*Haloarcula* sp. CBA1115
narG/nxrA	Contig-100_18_5	*Candidatus* Kuenenia stuttgartiensis	11	Similar to nitrate reductase subunit NarG	Q1PZD8	99	*Candidatus* Kuenenia stuttgartiensis
narG/nxrA	Contig-100_7_6	Unclassified bacteria	8	Uncharacterized protein	A0A0M2U6Y8	71	*Clostridiales* bacterium PH28_bin88
narH/nxrB	Contig-100_26_4	Unclassified bacteria	12	Uncharacterized protein	T0MN75	53	Candidate division *Zixibacteria* bacterium RBG-1
narH/nxrB	Contig-100_18_2	*Candidatus* Kuenenia stuttgartiensis	6	Strongly similar to nitrate reductase (NarH)	Q1PZD5	100	Candidatus *Kuenenia stuttgartiensis*
narH/nxrB	Contig-100_7_7	Unclassified bacteria	7	Chemotaxis protein CheY	A0A0M2U7X6	75	*Clostridiales* bacterium PH28_bin88
narI	Contig-100_51_2	Unclassified *Peptococcaceae*	7	Putative iron-sulfur-binding reductase	F7NDM4	64	*Acetonema longum* DSM 6540
nasA	Contig-100_1580_1	N/A [c]	100	Uncharacterized protein	X0UVK5	50	Marine sediment metagenome
nifD	Contig-100_75_2	*Desulfitobacterium hafniense*	4	Oxidoreductase, nitrogenase component 1	G9XNJ1	74	*Desulfitobacterium hafniense* DP7
nifH	Contig-100_0_18	Unclassified *Clostridia*	78	Putative multidrug resistance protein	D8WWN7	99	*Clostridia* bacterium enrichment culture clone BF
nifH	Contig-100_75_1	*Desulfitobacterium hafniense*	15	Putative nitrogenase iron protein	G9XNJ2	77	*Desulfitobacterium hafniense* DP7
nirK	Contig-100_276_2	*Desulfomonile tiedjei*	91	Uncharacterized protein	A0A0M2U6Z1	68	*Clostridiales* bacterium PH28_bin88
norB	Contig-100_4457_1	*Pontibacter* sp. BAB1700	58	Putative nitric oxide reductase	F8S994	74	Uncultured bacterium
norB	Contig-100_865_2	*Formosa* sp. AK20	26	Nitric oxide reductase	A0A0F2NRP9	96	*Flavobacteriales* bacterium BRH_c54
nrfA	Contig-100_77_1	*Candidatus* Kuenenia stuttgartiensis	39	Hypothetical (Triheme) protein	Q1Q0T9	99	*Candidatus* Kuenenia stuttgartiensis
nrfA	Contig-100_124_2	*Candidatus* Kuenenia stuttgartiensis	0	Cytochrome c family protein	Q1Q5N8	97	*Candidatus* Kuenenia stuttgartiensis
nrfH	Contig-100_3851_1	*Thermincola potens*	3	NapC/NirT cytochrome c domain-containing protein	A0A0L6W6D3	67	*Thermincola ferriacetica*
nosZ	contig-100_430_2	uncultured bacterium	100	Nitrous oxide reductase protein NosZ	I6ZZA8	74	Melioribacter roseus (strain JCM 17771 / P3M-2)
amoA/pmoA	Contig-100_1144_2	Unclassified *Nitrosomonas*	70	AmoA	E3UMY3	91	Uncultured bacterium

amoB/pmoB	Contig-100_8938_1	*Nitrosomonas eutropha*	18	Ammonia monooxygenase	A0A0F7KEV5	86	*Nitrosomonas communis*

[a] Based on uniprot May 11, 2016
[b] Contig-100 is the default IDBA_UD output for a kmer-run of 100, the following number is the contig number and last number is the gene number on that contig
[c] Not assigned. No taxonomy could be assigned, because either no relevant blast hit was found, or relevant blast hits were to diverse

Reference

1 Abu Laban, N., Selesi, D., Rattei, T., Tischler, P. & Meckenstock, R. U. Identification of enzymes involved in anaerobic benzene degradation by a strictly anaerobic iron-reducing enrichment culture. *Environ. Microbiol.* **12**, 2783-2796 (2010).

2 van der Waals, M. J. *et al.* Benzene degradation in a denitrifying biofilm reactor: activity and microbial community composition. *Appl. Microbiol. Biotechnol.* **101**, 5175–5188 (2017).
